# Progress in the use of ORS and zinc for the treatment of childhood diarrhea

**DOI:** 10.7189/09.010101

**Published:** 2019-06

**Authors:** Robert E Black

**Affiliations:** Bloomberg School of Public Health, Johns Hopkins University, Baltimore, Maryland, USA

Worldwide deaths from childhood diarrhea have declined in recent decades, but still total a half million per year [1]. A major contributor to this decline has been the use of oral rehydration solution (ORS) to both treat and prevent dehydration from diarrhea [2]. In fact, it was 50 years ago in 1968 that the scientific basis of glucose facilitated sodium absorption that led to ORS was first published [3,4]. Ten years later in 1978, the World Health Organization launched the global diarrheal control program with ORS at its heart [5], and ORS was hailed as “potentially the most important medical advance” of the 20th century [6]. After rapid scale-up of ORS use from 1980 with support from the World Health Organization (WHO) and other international agencies, ORS is now used for treatment in about a third of diarrhea episodes [7,8]. A second advance in diarrhea treatment was the demonstration that oral zinc would shorten the duration and severity of diarrhea [9,10]. The use of zinc for treatment of all childhood diarrhea was recommended by WHO and UNICEF in 2004 [11]; however, in spite of nearly all low-income and lower-middle income countries including zinc in their national diarrhea treatment guidelines, use in these countries has been low [7,8,12]. The opportunity for greater reduction in childhood diarrhea mortality if greater coverage of ORS and zinc for diarrhea treatment could be achieved has led to several calls for action [7,8,13].

Recent efforts to promote use of ORS and zinc for diarrhea treatment in some countries show that substantial progress is possible and provide important lessons for other countries. In six districts in the Indian state of Gujarat and twelve district of Uttar Pradesh, a pilot program, Diarrhea Alleviation through Zinc Therapy (DAZT), during 2011-2014 trained and supported public sector providers, made individual educational visits (”drug detailing”) to private providers, including the informal rural practitioners who provide the majority of care, and worked to ensure a supply of ORS and zinc at community level [14]. In the Gujarat districts, ORS use increased from 15% to 40% and use of both ORS and zinc increased from 2% to 18%; however, in the districts of Uttar Pradesh ORS use remained at only 20% and zinc use only increased to 7%.

The Clinton Health Access Initiative (CHAI) during 2012 to 2016 engaged with governments, heath care providers, and communities to improve diarrhea treatment in Kenya, Uganda and selected high-mortality states of India and Nigeria [15]. The CHAI program enhanced and scaled-up the DAZT program to the 22 districts in Gujarat and 39 districts of Uttar Pradesh, as well as the entire state of Madhya Pradesh. In particular, the program expanded the drug detailing to providers, established a more efficient and profitable supply chain for ORS and zinc and worked with the public sector in training and support of diarrhea treatment in health facilities and in the community [16]. Importantly, CHAI also implemented a multi-channel mass media campaign targeting caregivers, filling a gap in demand creation in the previous program. This program was able to increase combined ORS and zinc use to 28% in Gujarat, 32% in Madhya Pradesh, and to a lesser extent in Uttar Pradesh where combined use reached 12% (although ORS use did increase to 44%). The experiences in India show the value of integrated approaches targeting public and private sectors involving both creation of demand for treatment and supply thru trained and motivated providers and available commodities.

In the three African countries where the CHAI activities were implemented, ORS coverage at baseline ranged from 38% to 44% [15] but zinc use was negligible. The CHAI interventions included improving knowledge about appropriate treatment for caregivers via schools and community health workers and for providers via education and mentorship as well as drug detailing. Extensive work was needed to increase the availability of affordable ORS and zinc products and establish a functional supply chain, as well as assist with policies, such as approving zinc as an over-the-counter medicine and adding it to the national essential medicines list [17]. An important strategic activity in these countries was the change from separate ORS and zinc products to a co-packaged product, particularly in the public sector [15]. These efforts from 2012 to 2016 contributed to combined ORS and zinc coverage levels of 30% in the 8 Nigerian states, 30% in Uganda and 15% in Kenya at the end line assessment [15]. These coverage levels exceed those of most countries in Africa. This is likely the result of the successful implementation of the comprehensive interventions needed to scale up recommended diarrhea treatments, but especially influential in regard to zinc is the co-packaging with ORS that can quickly equalize their use.

Programs to accelerate interventions in partnership with countries, such as those implemented by CHAI in four countries, provide examples of what can be accomplished in a short time period and offer important lessons on what challenges need to be overcome in these and similar settings. These evaluations illustrate that countries can improve diarrhea treatment if they address product and supply issues, provider incentives, consumer demand and the policy environment. This work builds on an existing body of evidence in what works in scaling up zinc and ORS [18]. The question is no longer *what* needs to be done to increase the quality of childhood diarrhea treatment – it is *when* we will collectively as a global community take action on this evidence.

While progress was demonstrated in each setting where CHAI implemented, it is sobering to note that in no setting was ORS and zinc together used as recommended for treatment in more than a third of diarrhea episodes. While this progress likely averted a substantial number of deaths [15], higher coverage of appropriate treatment is needed in these and other high-burden countries to prevent most of the remaining childhood diarrhea deaths. We know what it takes to eliminate preventable diarrhea deaths in children globally. On this 50^th^ anniversary of the discovery of ORS, let us recommit ourselves to making this a reality.
